# Virtual Biopsy: Just an AI Software or a Medical Procedure?

**DOI:** 10.2967/jnumed.121.263749

**Published:** 2022-04

**Authors:** Jacob M. Murray, Bodo Wiegand, Boris Hadaschik, Ken Herrmann, Jens Kleesiek

**Affiliations:** 1 Institute for AI in Medicine, University Hospital Essen, Essen, Germany;; 2 Philips, Eindhoven, The Netherlands;; 3 Department of Urology, University Hospital Essen, Essen, Germany;; 4 German Cancer Consortium, University Hospital Essen, Essen, Germany;; 5 West German Cancer Center Essen, Essen, Germany;; 6 Department of Nuclear Medicine, University of Duisburg–Essen, Duisburg, Germany; and; 7 Cancer Research Center Cologne Essen, Essen, Germany

The term *virtual biopsy* is gaining traction. Since 2015, the number of publications referencing the concept in the search engine PubMed has doubled, and in 2021 it reached its highest level yet. This raises the question of what the unique characteristics of a virtual biopsy are and how it might be distinguished from other advances in computer-assisted medicine. For optimists, it may be the next step toward a less invasive, more personalized era in medicine that harnesses recent advances in functional imaging and artificial intelligence (AI) to generate patient management decisions. For skeptics, the term may ring hollow, like yet another marketing phrase within the hype surrounding AI in the medical field. Ultimately, the juxtaposition of *virtual* and *biopsy* contains within it not merely the possibility of adding another tool to the physician’s toolbox but the aspiration that the biopsy, a crucial procedure in the diagnosis of disease, may be transformed from physical to virtual while still providing diagnostic and prognostic information at least at the level of a traditional, physical biopsy, serving as reference standard. We assess the current state of this aspiration and conclude that although hurdles remain, the virtual biopsy is poised to replace the physical biopsy as a central step in the diagnosis and management of certain diseases.

Its origin from the Greek words *bios* for life and *opsis* for sight illustrates that a biopsy makes information that is relevant to biologic existence available for insight. This has traditionally occurred via the direct visual examination of invasively retrieved tissue specimens by a pathologist under a microscope. However, the term *virtual biopsy*—in analogy to the more commonly encountered liquid biopsy—suggests that what is relevant is not the type of specimen or direct visual appreciability but the usefulness and accuracy of the biologic insight that can be gleaned from the biopsy procedure. Interestingly, the term *virtual,* as describing something simulated, might be considered a misnomer here, since the radiologic images that serve as the inputs of a virtual biopsy reflect physical realities in the same way histology slides might. We stipulate that the validity of a virtual biopsy as a form of biopsy procedure, and as opposed to a piece of software, is determined by the quality and completeness of the medically relevant information it can deliver, even in excess of the imperfect physical biopsy reference standard. In particular, whereas the image resolution available in microscope-based analysis of physical biopsy samples will not be available in virtual biopsies, the volume of imaged tissue is larger, that is, the complete tumor manifestation of *primarius* and its *filiae* in a full-body PET exam. This may allow for a different, potentially more complete molecular phenotype characterization of disease—for example, addressing the topic of tumor heterogeneity. There is also hope that using advanced AI techniques, previously unavailable information can be extracted. For example, there are super-resolution approaches to enhancing resolution. But there are also efforts that attempt to see beyond—that is, to extract new information by identifying nonlinear relationships and to make it available to humans. This, although not yet a reality, could then allow for inference of genetic traits from phenotype. Furthermore, the validity of a virtual biopsy will depend on whether the provided information is accepted as a foundation of medical management by the various stakeholders, such as patients, physicians, insurance companies, and regulatory bodies. The acceptance of virtual biopsies by insurance companies and regulatory bodies will then also determine the status of virtual biopsies as procedures in the context of billability and health economics.

Currently, virtual biopsies are explored primarily within the research setting. Multiple areas of application in oncology are emerging. Morawitz et al. assessed the ability of 4 different imaging modalities in determining axillary lymph node status in women diagnosed with breast cancer. Comparing the imaging modalities with histopathology as a reference standard, they concluded that if both ^18^F-FDG PET/MRI and sonography are positive, “one might consider dispensing with axillary histopathologic sampling” (*1*). Although the term *virtual biopsy* is not explicitly mentioned, the raised possibility of foregoing physical biopsy places the work within this context. For ovarian cancers, an entity with broadly intractable survival rates, Martin-Gonzalez et al. proposed a combination of quantitative imaging features and genomics markers to monitor therapy and improve patient management (*2*). For lung cancer, which remains the leading cause of cancer-related mortality, the introduction of targeted and immunotherapies has dramatically altered the treatment landscape and survival rates. Consequently, the identification of targetable mutations and expression levels has become a crucial step in patient management and constitutes a further reason, apart from determining the histologic cancer subtype, for performing a physical tissue biopsy. In this context, Wen et al. linked non–small cell lung cancer radiomics features and clinical markers to estimate expression levels of immunotherapy target proteins and to potentially inform patient management decisions without the need for physical biopsy (*3*). Taken together, this work highlights that virtual biopsies are being investigated for some of the most prevalent oncologic entities. Prostate cancer, too, belongs to this group.

The diagnostic algorithm for prostate cancer is changing to accommodate a more pronounced role of imaging in decisions surrounding physical biopsy. Prostate cancer is the most common solid cancer in men but presents with various forms of aggressiveness, and overdiagnosis of indolent disease causes more harm than benefit. As with other oncologic entities, a common aim of research that falls within the realm of virtual biopsy is to establish a robust link between radiologic imaging features and histopathology in terms of the presence or absence of clinically significant prostate cancer. In this context, Eklund et al. showed that the addition of MRI-targeted biopsy allows omission of a significant number of biopsies and reduces the detection of insignificant prostate cancer in performed biopsies in men undergoing prostate cancer screening (*4*). By combining multiparametric MRI with prostate-specific membrane antigen PET/CT imaging, the need for physical biopsy in patient management might disappear altogether. Emmett et al. investigated 291 patients for whom MRI, pelvic prostate-specific membrane antigen PET, and systematic or targeted transperineal biopsy were performed and concluded that on the basis of the high detection rate of clinically significant prostate cancer and the high negative predictive value, “further randomised studies will determine whether biopsy can safely be omitted in men with high clinical suspicion of [clinically significant prostate cancer] but negative combined imaging”(*5*). This conclusion is underscored further by a recent retrospective case series published by Meissner et al. including 25 patients who underwent radical prostatectomy without prior biopsy, based solely on MRI and prostate-specific membrane antigen PET results. All 25 patients showed clinically significant prostate cancer in the postsurgical histopathology examination (*6*). These results lay the scientific foundation for clinical trials examining virtual biopsies to change the diagnostic pathways in prostate cancer care. However, despite increased scientific exploration of topics that fall within the scope of virtual biopsies, few discoveries have so far bridged the divide between academia and industry.

Current Food and Drug Administration–cleared software tools are focused largely on providing decision support and decision automation within a diagnostic framework that continues to rely on physical biopsy.

On the one end of the diagnostic cascade, for instance, there are Food and Drug Administration–cleared solutions that aim to optimize the prostate MRI workflow through custom AI-augmented software before the extraction of a physical biopsy, such as Quantib (https://www.quantib.com/). On the other end of the cascade, there are Food and Drug Administration–approved AI tools to detect prostate cancer after the biopsy has been performed and the tissue sample is available for analysis, such as Paige (https://www.paige.ai/). In both cases, the software can be understood as a diagnostic tool or decision support system. They do not, however, directly replace the physical biopsy itself. Instead, they might indirectly influence management by providing evidence that a physical biopsy can safely be avoided. A medical procedure in the context of a virtual biopsy would refer to a software tool that provides a diagnosis of a histologic type without the need to perform an invasive biopsy. Whether this becomes possible depends on the density and extractability of the information that serves as input to these software tools. If imaging and clinical data were to contain a sufficient level of information that can effectively be used by machine learning techniques to accurately classify disease, then patient management without the need for a physical biopsy could become a reality. Because a virtual biopsy is not restricted to the actual target of a physical biopsy, patient management might even be improved in the future.

Defining and attaining the required level and reliability of information that is accessible through virtual biopsy to obtain stakeholder acceptance remain the central hurdle on the path toward virtual biopsies. As Penzkofer et al. pointed out, “a change in indication from a ‘radiological diagnosis support tool’ to ‘clinical decision-making tool’ would need to undergo rigorous testing against clinically valid endpoints, such as the presence of clinically significant lesions in positive and negative cases” (*7*). This implies that what is ultimately required are large-scale prospective randomized controlled trials providing evidence that virtual biopsies can serve as substitutes for real biopsies. In addition, assessments of health-economic effects (in terms of potential cost reductions) and of quality-of-life changes (in terms of avoidance of complications associated with a physical biopsy) that will result from switching to virtual biopsies are needed. Then, stakeholders, such as patients, individual physicians, and guideline-issuing professional societies, as well as regulatory bodies, might begin to adopt virtual biopsies as medical procedures with management-determining results.

The final component that will determine the future of virtual biopsies is acceptance by payers and the availability of a billing code for reimbursement. Health-care systems are nearing their breaking point, which has prompted regulators and budget holders to look critically at how medical devices and solutions benefit patients. Value-based health care becomes more and more important, whereby reimbursement is based on patient health outcomes instead of the volume of services provided by health-care professionals. Additionally, health technology assessment bodies are developing strategies to evaluate the new digital technologies entering the market. A case in point is the United Kingdom, where evidence of clinical effectiveness and economic value must be generated in accordance with the Evidence Standards Framework for Digital Health Technologies (*8*) outlined by the National Institute for Health and Care Excellence. Digital health-care technologies are becoming increasingly popular in other markets as well, and budget holders, payers, and providers are beginning to see their importance. As an example, the law on prescription and reimbursement of digital health technologies in Germany governs how statutory health insurances compensate digital health applications. Still, manufacturers must demonstrate to the Federal Institute for Drugs and Medical Devices that their solutions lead to positive health outcomes. The new law improves the legal status of digital health apps, which are considered low-risk medical devices. The future of high-risk health applications remains open. To establish virtual biopsies as medical procedures, in-depth consideration of their role within the low-risk to high-risk medical device spectrum will be necessary.

In conclusion, virtual biopsies are at the center of a flourishing and growing research effort. But the ascent of virtual biopsies is not limited to academia. Despite regulatory and reimbursement challenges that remain, virtual biopsies present the logical next step in patient care and thus resemble a very likely future development for the diagnosis and management of certain diseases.

## DISCLOSURE

No potential conflict of interest relevant to this article was reported.
